# Brugada Syndrome: Focus for the General Pediatrician

**DOI:** 10.3390/children11030281

**Published:** 2024-02-25

**Authors:** Alessia Speranzon, Daniela Chicco, Paolo Bonazza, Raffaele D’Alfonso, Marco Bobbo, Biancamaria D’Agata Mottolese, Egidio Barbi, Thomas Caiffa

**Affiliations:** 1 Department of Medical, Surgical and Health Sciences, University of Trieste, 34127 Trieste, Italy; alesperanzon@libero.it (A.S.); egidio.barbi@burlo.trieste.it (E.B.); 2 Institute for Maternal and Child Health, IRCCS “Burlo Garofolo”, 34127 Trieste, Italy; daniela.chicco@burlo.trieste.it (D.C.); marco.bobbo@burlo.trieste.it (M.B.); biancamaria.dagatamottolese@burlo.trieste.it (B.D.M.); 3 General Pediatrician, 58100 Grosseto, Italy; paolo.bonazza@gr.omceo.it (P.B.); raffaele.dalfonso@gr.omceo.it (R.D.)

**Keywords:** Brugada syndrome, sudden cardiac death, Brugada pattern, arrhythmia, pediatric population

## Abstract

Brugada Syndrome is an “inherited” channelopathy characterized by a predisposition to syncope and sudden death. It typically presents in young adults but is also known to affect the pediatric population, even if the prevalence is low compared to the adult population. The diagnostic ECG pattern shows coved-type ST-segment elevation in the right precordial leads, occurring spontaneously or after provocative drug tests with IV administration of Class I antiarrhythmic drugs. However, the electrocardiographic findings may vary, and transient or concealed forms of the syndrome further complicate diagnosis, necessitating thorough evaluation and close clinical follow-up. The clinical presentation of Brugada Syndrome may range from asymptomatic individuals to patients who have experienced syncope or sudden cardiac arrest. The syndrome remains underdiagnosed due to its elusive symptoms and the absence of abnormal findings between episodes. Additionally, specific triggers such as fever, certain medications and alcohol consumption may unmask the electrocardiographic changes and provoke arrhythmias in susceptible individuals. Given its elusive nature, early diagnosis and risk stratification are crucial in identifying individuals who may benefit from an implantable cardioverter defibrillator, the mainstay of treatment for high-risk patients, or pharmacological interventions.

## 1. Introduction

Brugada Syndrome is a genetic disease with autosomal dominant transmission, belonging to the group of channelopathies, characterized by a pathognomonic alteration of the ECG (Brugada ECG pattern), which consists of a coved-type ST-segment elevation and T wave inversion in at least one of the right precordial leads [1].

It is an arrhythmogenic disease linked to an alteration of Na^+^ channels, associated with an increased risk of sudden cardiac death due to the potential onset of ventricular tachycardia or fibrillation in individuals with a structurally healthy heart.

However, in recent years, several studies have highlighted how mutations in genes that encode for potassium and calcium channels can also be involved in the pathogenesis of the disorder [2].

Brugada Syndrome is responsible for 4–12% of all cases of sudden cardiac death and of 20% of sudden cardiac death cases in individuals with a structurally normal heart [2]; in addition, it is estimated that a significant percentage of sudden infant death syndrome can be attributed to pathologies due to alteration in the sodium channel, including Brugada Syndrome [3].

Since the finding of an altered ECG is a fundamental requirement for the diagnosis, it is not easy to estimate the real prevalence of the pathology since a significant proportion of the affected patients is asymptomatic. Several studies have estimated the prevalence to be between 1:2000 and 1:5000 [3]. Another confounding factor is the variability and intermittence of the typical ECG signs, making it difficult to recognize the affected subjects.

Typically, Brugada Syndrome occurs in males between the third and fourth decades of life, while the onset in the pediatric age is rarer [4]; moreover, in the pediatric population, no substantial difference between male and female sex has been highlighted.

This difference in the manifestation of symptoms can be explained by the influence of sex hormones: estrogen, progesterone and testosterone. Testosterone increases the risk of atrial arrhythmias due to increased adrenergic activity [5,6,7]; while in some case reports, a normalization of ECG following orchiectomy was reported [3].

The lower incidence in females in the adult age group compared to girls/adolescents may be due to the influence of female hormones in reducing the expression of K^+^ channels at the level of epicardial cells in the right ventricle [7].

## 2. Materials and Methods

For the sake of this narrative review, we scanned the currently available literature. Data were identified through searches of MEDLINE, UpToDate, Google Scholar and references from relevant articles. The following search string was used for MEDLINE: (“Brugada syndrome in children” OR “pediatric Brugada Syndrome”). 

Over 400 articles were initially found through this search strategy, which included articles published between 2000 and 2023 in English and other languages, provided that an English abstract was available. 

We prioritized reviews and the peer-reviewed literature. The examination of the literature was focused on the pediatric population. However, when considering broader concepts applicable to both pediatric and adult patients, the search was not restricted solely to the pediatric literature. We then integrated this information with our pediatric experience.

## 3. Pathophysiology and Genetics

The genetic basis of Brugada Syndrome has been demonstrated since 1998, when an association between symptoms and a loss of function of the SCN5A gene, present in 20–25% of patients, was described [8]. This gene codes for the α subunit of the Na^+^ channel at the level of cardiac cells, which plays a fundamental role in the generation of the action potential and the propagation of the electrical impulse in the heart [1]; the Na^+^ channel defect determines, at the cellular level, a marked shortening of the action potential confined exclusively to the epicardial layers of the right ventricular outflow tract (RVOT), where outgoing K^+^ channels are concentrated [9].

It explains the alterations in leads V1–V3 and the possibility, in some cases, of recording them only by positioning the electrodes in the second or third intercostal spaces, i.e., in the area directly facing the outflow tract of the right ventricle [10].

Minimal ultrastructural changes, such as increased collagen and subepicardial fibrosis and decreased gap junctions, have been described in the area of the arrhythmogenic site. The meaning to be attributed to these ultrastructural alterations is still uncertain [6]. 

Over the years, several other genes mutated in Brugada Syndrome patients have been identified: some coding for Na^+^ channels, some for K^+^ channels, and others for Ca^2+^; however, only variants of the SCN5A gene are considered pathogenic to date. In each case, the result is a reduction in the Na^+^ and Ca^2+^ input current or the K^+^ output current [1].

Furthermore, over the years, the idea has been that Brugada Syndrome can follow a more complex polygenic inheritance: it could result from the co-presence of several common variants conferring susceptibility in each individual [11].

The confirmation of the fact that the role of genetics in Brugada Syndrome is not yet fully understood comes from the fact that, at the time of diagnosis, a known genetic mutation is detected only in less than 30% of cases.

## 4. Clinical Presentation 

Brugada Syndrome occurs mainly in adulthood, while its detection in children is rare.

The main symptoms include the following:

- Cardiac syncope: a differential diagnosis is required with the other causes of syncope, with vasovagal syncope first;
- Palpitations;
- Nocturnal agonal respirations;
- Cardiac arrest;
- Sudden cardiac death.

These symptoms may result from the onset of polymorphic ventricular tachycardia or ventricular fibrillation; presentation with monomorphic ventricular tachycardia is described in the literature, usually in subjects carrying a mutation of the SCN5A gene, but it is a rare occurrence that should lead to the exclusion of the other pathologies such as arrhythmogenic cardiomyopathy of the right ventricle [12].

When the diagnosis is made, 30–35% of patients are symptomatic, with a prevalence of syncope rather than cardiac arrest and sudden death, while the remaining 70–75% of cases are asymptomatic.

In the pediatric population, the most common initial presentation of Brugada Syndrome is a family history positive for Brugada Syndrome (47%), followed by the incidental finding of the ECG pattern (25%), syncope (14%), arrhythmia (13%) and sudden cardiac death (1%) [13]. Other initial manifestations in children include sinus node dysfunction, atrial arrhythmias (including atrial fibrillation and flutter) and interventricular conduction disturbances [14,15].

It is also known that the onset of symptoms is favored by some conditions such as fever, some drugs, large meals and alcohol abuse; moreover, they very often occur in moments of vagal hypertonicity, at night or at rest rather than during physical activity [1,3]. This latter feature differentiates them from ventricular arrhythmias triggered by an adrenergic stimulus, as, for instance, in cathecolaminergic polymorphic ventricular tachycardia (CPVT). In athletes, the syncopal crisis can occur at the end of a physical effort in which there is a simultaneous reduction of the adrenergic tone and an increase of the vagal tone.

## 5. Diagnosis

**Figure 1:**
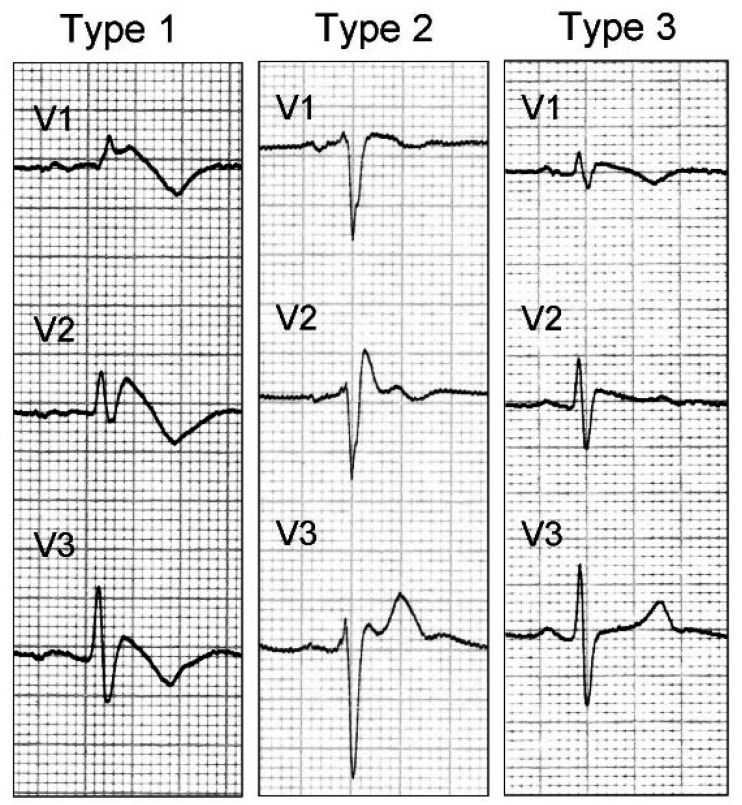
The different morphologies of the Brugada pattern [16].

The diagnosis of Brugada Syndrome is based on the ECG finding of the Brugada pattern in at least one of leads V1–V3. Affected individuals may have three different ECG patterns, but only type 1 is diagnostic (Figure 1).

- Type 1: characterized by elevated J point and ST ≥ 2 mm, coved, which typically has a descending course, followed by negative T wave.
- Type 2: characterized by elevated J point and elevated ST ≥ 1 mm, saddle-shaped, flat and followed by positive T wave.
- Type 3: indicated by J point and ST elevation < 1 mm.

The criteria for making the diagnosis of Brugada Syndrome are as follows:

- Spontaneous or drug-induced type 1 ECG pattern;
- Type 2 or type 3 ECG pattern when, after a provocation test, a type 1 ECG pattern is induced [11].

**Figure 2:**
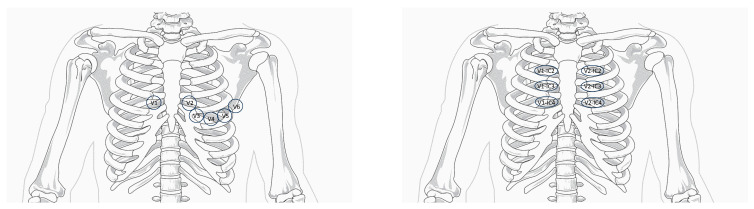
(**Left**) Standard-lead electrocardiogram positions. (**Right**) High V1–V2 position. Modified from Krahn A. et al., “Brugada Syndrome” [1].

Since the pathophysiological substrate seems to be located at the level of the right ventricular outflow tract, to increase the sensitivity of the ECG, it may prove necessary to place leads V1–V2 in the second and third intercostal space parasternal right and left, i.e., in correspondence with the RVOT [1] (Figure 2).

It should be emphasized that “type 1 Brugada pattern” and “Brugada syndrome” should not be considered the same thing. In fact, if syncope, history of cardiac arrest, sudden death or other suspicious symptoms are not reported in clinical history, we should refer to “Brugada pattern” [17]. This distinction is fundamental for a correct stratification of the arrhythmic risk and, consequently, for an appropriate management of the patients since the therapies currently available for this disease are not without side effects.

Although the diagnosis necessarily requires the presence of pattern 1 on the ECG, we can use the Shangai score (Table 1), which attributes a specific score to each sign and symptom, from the sum of which a diagnosis can be hypothesized as probable/certain (score ≥ 3.5), possible (score 2–3) or non-diagnostic (<2) [8].

When basal ECG is not diagnostic but there is a strong clinical suspicion, the execution of the provocation test with Na^+^ channel blocking drugs may prove helpful: the most effective in uncovering a type 1 ECG pattern are Ajmaline, Flecainide and Procainamide. A consensus has not yet been established on the most suitable age for children to perform this test, nor on the safety profile of the drugs used. However, we have noted that the outcome may vary over time and that retesting after puberty can unmask the Brugada pattern in patients who test negative before puberty.

Given the potential risk of adverse events of challenging testing with blockers of the Na^+^ channel and considering the low diagnostic yield at pediatric age [8], it seems reasonable not to perform this test before the age of 12 to 15 years [4]. 

Contraindications to performing the test are as follows:

- Baseline ECG showing type 1 pattern (no benefit would be gained, since diagnosis is already clear, while potentially risking VT/VF induction);
- Lengthening of the PR segment on the baseline ECG due to the risk of inducing an AV block [11].

Furthermore, since, in children, the type 1 pattern is more easily induced by temperature variations, the execution of an ECG during fever is recommended in cases of non-diagnostic basal ECG but with a clinical suspicion of the disease. Hyperthermia can increase the action potential difference between the epicardium and the endocardium, playing an essential role in arrhythmogenesis. Another possible explanation is that fever can reduce the function of Na^+^ channels, thus unmasking Brugada ECG pattern [2].

Before making the diagnosis of Brugada Syndrome, it is necessary to exclude other causes of ST segment elevation. Among these, we recognize the following:

- Arrhythmogenic right ventricular cardiomyopathy: in this case, ECG abnormalities are not intermittent but are always detectable.
- Early repolarization syndrome, a condition that is not infrequently found in highly trained young subjects and which is, in most cases, a benign condition.
- Right bundle branch block.
- Acute myocardial ischemia or infarction.
- Mechanical compression of the right ventricle, for example, in subjects with pectus excavatum.
- Electrolyte abnormalities (hyperkalemia, hypercalcemia): in the case of symptoms or suspicious changes on the ECG, it is recommended to carry out a blood test to look for electrolyte alterations and, if present, correct them to avoid the risk of arrhythmias [17].

In clinical practice, in the case of an ECG with dubious characteristics, it is useful to repeat the ECG with high V1–V2 leads position. 

## 6. Risk Stratification

Although much progress has been made in understanding Brugada Syndrome, we still do not have sufficient indications to predict the risk of potentially fatal arrhythmias. We need to consider the type of clinical presentation, ECG pattern and inducibility of arrhythmias upon testing during an electrophysiological study.

Such a study would verify the predisposition to develop malignant arrhythmias through programmed ventricular pacing. It can identify subjects at high risk of arrhythmic events. Since the predictive power of this test is still debated, it is not easy to select the patients most likely to benefit from it. 

In a recent study, Gaita et al. found that patients with a spontaneous type 1 pattern associated with a positive electrophysiological study were at higher risk of developing malignant arrhythmias compared to subjects with an ECG Brugada pattern but a negative electrophysiological study (0.4% per year) [18]. 

An electrophysiological study could be considered in asymptomatic subjects with a spontaneous type 1 ECG pattern as risk stratification, while it is not recommended in symptomatic subjects (high-risk patients) and asymptomatic patients with drug-induced type 1 ECG pattern only (low-risk patients) [17].

Individuals at the highest risk of developing malignant arrhythmias are, in addition to those who have experienced cardiac arrest [2], those with a spontaneous type 1 ECG pattern and a history of cardiogenic syncope (risk/year 2.3–3.7% [1]).

Individuals with multiple risk factors are at moderate–high risk of arrhythmias. Among the risk factors acknowledged in adult Brugada population, we can mention the following:

- Family history of sudden cardiac death at an early age (first degree relative < 35 years);
- History of syncope of cardiac origin;
- Male sex;
- Inducibility of malignant arrhythmias during an endocavitary electrophysiological study;
- Presence of fragmented QRS at ECG;
- Coexistence of atrial fibrillation;
- Coexistence of early repolarization in inferolateral leads without ST-segment elevation;
- Associated ventricular conduction disturbance;
- The appearance of a type 1 ECG pattern or the increase in ST-segment elevation during the recovery phase of the stress test.

Asymptomatic subjects with drug-induced type 1 Brugada pattern, in the absence of the risk factors listed above, have the same risk as the general population (0.5% [10]) of developing life-threatening arrhythmias; in these cases, however, a close follow-up of the first-grade family members is necessary to assess the potential presence of familial Brugada Syndrome [19,20,21].

Of note, despite several studies showing a higher prevalence of SCN5A mutation in symptomatic patients than in asymptomatic ones, to date, there has been no strong evidence that the presence of a mutation is associated with increased arrhythmic risk [7,11,22].

Reports of new genes associated with the syndrome are constantly increasing, even if, for many genes, the pathogenetic significance is uncertain; even with these limitations, genetic counseling should always be part of the diagnostic process of Brugada Syndrome in the hope of being able to use genetic information in the future both for risk stratification and for family screening and counseling.

Focusing on the pediatric population, the manifestation of Brugada Syndrome before 12 months of life seems related to a higher risk of potentially fatal arrhythmias. In a recent case report and literature review regarding this group of patients, authors highlighted two important aspects:

1. A total of 59% of the subjects presented a type 1 ECG pattern at the time of the first manifestation (higher percentage compared to the entire affected population), with more than 30% of infants having other associated conduction abnormalities at ECG;
2. More than 90% of children had positive genetics for mutations in the SCN5A gene, compared to approximately 20–25% in the general population of affected patients. Moreover, many of these patients had more than one mutation in this gene on genetic analysis [23].

## 7. Treatment

The therapeutic approach depends on the patient’s risk of developing life-threatening arrhythmias, it is advisable to perform a close follow-up in these patients to identify any new arrhythmic risk factors early.

Recommendations for the treatment of Brugada Syndrome are generally extrapolated from the adult population, with often weak or virtually absent indications for pediatric age. It must be underlined that arrhythmic risk before 12–15 years of age is generally low [7]; therefore, treatment recommendations should be weighted case by case, not forgetting the possible serious adverse events related to some therapies (i.e., potential arrhythmic risk of quinidine or possible complications of an implantable cardioverter defibrillator, like inappropriate shocks and infections). 

All patients diagnosed with a Brugada-type ECG pattern are recommended to take a series of precautions:

- Treat fever early with antipyretics, especially in children who are more vulnerable to increased body temperature than adults, particularly in the 0–5 years age group [24]; preventive treatment with Paracetamol is recommended before carrying out the common vaccinations [8].
- Avoid drugs that can promote the onset of symptoms and induce the Brugada pattern on the ECG. Among the drugs to avoid absolutely, we mention the following:
  ○ Antiarrhythmics such as ajmaline, flecainide, procainamide, propafenone, allapinine, ethacizine and pilsicainide.
  ○ Psychotropics such as amitriptyline, clomipramine, desipramine, lithium, loxapine, nortriptyline, oxcarbazepine and trifluoperazine.
  ○ Anesthetics/analgesics such as bupivacaine, procaine and propofol. The lidocaine used for local anesthesia does seem to be safe if the quantity administered is low and if it is administered in combination with adrenaline (local action).
  ○ Other substances such as acetylcholine, cannabis, cocaine and ergonovine.

When managing a patient with Brugada Syndrome, it is always good to consult the list of all drugs to avoid, available at www.brugadadrugs.org (accessed on 1 November 2023) [3].

- Avoid large meals with a high content of carbohydrates and alcohol abuse [11].

In asymptomatic patients in whom the Brugada pattern on ECG is found only after drug induction, these measures are usually sufficient to avoid the risk of arrhythmias [1], with a very low risk of developing symptoms such as malignant arrhythmias or sudden cardiac death (0.03% per year in a recent study) [18].

The only treatment effective in controlling ventricular arrhythmias and preventing sudden cardiac death is the implantation of the cardiac defibrillator. However, the use of this device is burdened by numerous adverse effects such as inappropriate shocks, malfunction of the electrodes, risk of infections resulting in the need for removal and substitution of the device, and the difficulty of acceptance by the patient [1,17].

An implantable cardioverter defibrillator is strongly recommended (Class of Recommendation I, Level of evidence A) in secondary prevention (patients with a history of cardiac arrest), as these subjects are at high risk of developing further malignant arrhythmic events. In subjects with one or more major risk factors, especially with a history of syncope of probable cardiogenic origin, the implantation of a cardiac defibrillator in primary prevention is recommended in class IIA [19].

The decision to implant a cardiac defibrillator should be made in consultation with the patient and the family, considering the specific characteristics and individualizing it based on clinical presentation, family history and genetic and family preferences [25].

Pharmacological therapy may include using quinidine, a class I antiarrhythmic, which can normalize the ECG tracing and reduce the risk of VF and sustained VT. However, it has a pro-arrhythmic potential and significant dose-related side effects (diarrhea, thrombocytopenia, anemia, neurological effects), which make its long term use difficult [1,11].

For these reasons, quinidine can be considered in patients who experience numerous arrhythmic episodes despite the implantation of the defibrillator, in patients who have denied it or in patients in whom it is contraindicated. In the pediatric population, the evidence of efficacy and safety in administering quinidine is still limited [2,8].

In patients with persistent arrhythmic episodes despite optimized drug therapy or in those intolerant to quinidine, radiofrequency ablation may be considered to eliminate the arrhythmogenic substrate located at the RVOT [26]. Although the incidence of life-threatening arrhythmias in these patients is relatively low, educating family members on resuscitation maneuvers and inviting them to attend a first aid course is of fundamental importance.

## 8. How to Manage Sports

Ventricular arrhythmias typically arise at rest or in conditions of bradycardia, which has given rise to doubts about the possibility of practicing sports in these patients. 

Arrhythmias are generally rare at a pediatric age [27] and even because children usually practice low-demand sports compared to adults. The benefits of regular physical activity at any age, especially during childhood and adolescence, must not be forgotten. Therefore, physical activity restrictions in children with Brugada Syndrome is usually less limiting compared to adults. 

According to the Italian protocols of Sports Medicine [21] (not only focused on the pediatric population), competitive sports eligibility can be granted in the following:

- Asymptomatic subjects with type 2 or type 3 patterns in the absence of a family history of sudden cardiac death or other risk factors (Class of Recommendation I Level of Evidence C);
- Subjects with type 1 pattern, without other risk factors, with a negative electrophysiological study (Class of Recommendation I Level of Evidence C).

Eligibility may also be reasonable in asymptomatic subjects with a spontaneous type 1 pattern at low risk (without family history of sudden cardiac death and/or Brugada Syndrome, etc.) (Class of Recommendation II Level of Evidence C).

Eligibility should be denied in patients with an uncertain high-risk score (see Sieira and Shangai scores), except in selected cases after an evaluation in highly experienced centers (Class of Recommendation II Level of Evidence C).

Eligibility should also be denied in symptomatic patients with a history of cardiac syncope or previous cardiac arrest with type 1 Brugada pattern (spontaneous or drug-induced) (Class of Recommendation III Level of Evidence B).

The significance of a positive electrophysiological study in asymptomatic subjects with type 1 pattern and without other risk factors remains controversial.

According to the Italian recommendations for the management of pediatric patients under the age of 12 with Brugada Syndrome (suspected or manifest) [2], sport suspension is not recommended in the following cases:

- Children with type 1 Brugada pattern found on the ECG during fever or as drug-induced without other risk factors for life-threatening arrhythmias.
- Genotype-positive/phenotype-negative children without a family history of sudden cardiac death or other risk factors.

It must be underlined that sport ineligibility (linked to a theoretical risk) does not correspond to the indication for an implantable cardioverter defibrillator or other interventional procedures. Neither an implantable cardioverter defibrillator implantation nor ablation should be performed for the sole purpose of achieving sport suitability [21].

**Figure 3:**
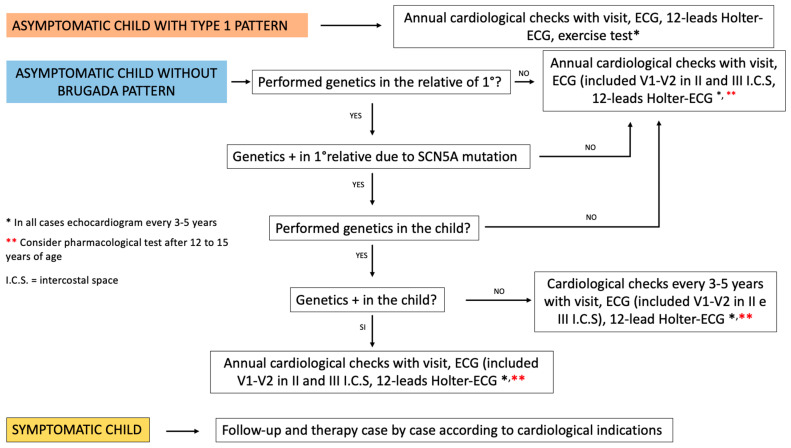
Modified from Andorin et al., “Impact of clinical and genetic findings on managing young patients with Brugada syndrome” [28].

Management proposal of pediatric patients with a first degree relative with BS (Figure 3).

## 9. Conclusions

In this review, we provide an overview on the diagnosis and management of patients with Brugada syndrome and their relatives with a particular focus on the pediatric population. As clearly observed, many issues still remain far from being clarified, with main regard to risk stratification and new therapies, especially in children [29,30]. 

In order to give practical advice to clinicians to reduce the misdiagnosis of Brugada Syndrome, we recommend the following:

- Repeat the ECG in children of patients with a first-degree relative with Brugada Syndrome at follow-up evaluations, since it can be dynamic and falsely negative;
- Execute an ECG with a high lead position (i.e., V1–V2 in the second and third intercostal spaces) at follow-up evaluations;
- Execute an ECG during fever;
- Remember the importance of family history, especially sudden cardiac death or major arrhythmias in first-degree relatives;
- Underline the importance of continuous follow-up over time, especially after puberty and in adult life, since arrhythmic events, although rare, mostly occur many years after diagnosis.

## Figures and Tables

**Table 1:** Shangai score.

	Points
**ECG findings:** (A) Spontaneous type 1 ECG	**3.5**
(B) Fever-induced type 1 ECG	**3**
(C) Type 2-3 ECG that converts to type 1 with provocation test	**2**
**Clinical hystory:** (A) Unexplained cardiac arrest or documented VF/polymorphic VT	**3**
(B) Nocturnal agonal respirations	**2**
(C) Suspected arrhythmic syncope	**2**
(D) Syncope of unclear etiology	**1**
(E) AF/flutter age < 30 years without clear etiology	**0.5**
**Family history:** (A) First- or second-degree relative with definite Brugada Syndrome	**2**
(B) Suspicious sudden cardiac death (fever, nocturnal, Brugada-aggravating drug) in a first- or second-degree relative	**1**
(C) Unexplained sudden cardiac death age <45 years in first- or second-degree relative with negative autopsy	**0.5**
**Genetic test:** (A) Probable pathogenic mutation in Brugada Syndrome susceptibility gene	**0.5**
Diagnostic criteria: probable/definite ≥ 3.5 points, possible 2–3 points, nondiagnostic < 2 points	
